# Longer pre-hospital delay in first myocardial infarction among patients with diabetes: an analysis of 4266 patients in the Northern Sweden MONICA Study

**DOI:** 10.1186/1471-2261-13-6

**Published:** 2013-01-29

**Authors:** Karin Hellström Ängerud, Christine Brulin, Ulf Näslund, Mats Eliasson

**Affiliations:** 1 Cardiology, Heart Centre and Department of Nursing, Umeå University, Umeå, Sweden; 2 Department of Nursing, Umeå University, Umeå, Sweden; 3 Cardiology, Heart Centre and Department of Public Health and Clinical Medicine, Umeå University, Umeå, Sweden; 4 Internal Medicine, Sunderbyn, Luleå, and Department of Public Health and Clinical Medicine, Umeå University, Umeå, Sweden

**Keywords:** Myocardial infarction, Diabetes mellitus, Pre-hospital delay, Sex differences

## Abstract

**Background:**

Reperfusion therapy reduces both morbidity and mortality in myocardial infarction, but the effectiveness depends on how fast the patient receives treatment. Despite the time-dependent effectiveness of reperfusion therapy, many patients with myocardial infarction have delays in seeking medical care. The aim of this study was to describe pre-hospital delay in a first myocardial infarction among men and women with and without diabetes and to describe the association between pre-hospital delay time and diabetes, sex, age, symptoms and size of residential area as a proxy for distance to hospital.

**Methods:**

This population based study was based on data from 4266 people aged 25–74 years, with a first myocardial infarction registered in the Northern Sweden MONICA myocardial infarction registry between 2000 and 2008.

**Results:**

The proportion of patients with delay times ≥ 2 h was 64% for patients with diabetes and 58% for patients without diabetes. There was no difference in delay time ≥ 2 h between men and women with diabetes. Diabetes, older age and living in a town or rural areas were factors associated with pre-hospital delay times ≥ 2 h. Atypical symptoms were not a predictor for pre-hospital delay times ≥ 2 h, OR 0.59 (0.47; 0.75).

**Conclusions:**

A higher proportion of patients with diabetes have longer pre-hospital delay in myocardial infarction than patients without diabetes. There are no differences in pre-hospital delay between men and women with diabetes. The largest risk difference for pre-hospital delay ≥ 2 h is between women with and without diabetes. Diabetes, older age and living in a town or rural area are predictors for pre-hospital delay ≥ 2 h.

## Background

Cardiovascular disease is the most common cause of death in adults with diabetes [1]. Although mortality from myocardial infarction (MI) has declined among patients with diabetes, it is still elevated compared to patients without diabetes, and women with diabetes have poorer outcome than men with diabetes [1-3].

Reperfusion therapy reduces both morbidity and mortality in MI, but the effectiveness depends on how fast the patient receives treatment. The greatest benefits occur when the patient has little pre-hospital delay and little in-hospital delay until receiving treatment [4], and the most effective treatments are started within one hour after symptom onset -- “the golden hour” [5]. Despite the time-dependent effectiveness of reperfusion therapy, more than half of the patients with MI have delays in seeking medical care by more than 2 hours from symptom onset, and pre-hospital delay times have been constant for several years [6,7].

Previous research has associated longer pre-hospital delay with socio-demographic factors such as old age, female sex [8], low educational level [9,10] and clinical factors such as heart failure and previous MI [7]. However, conflicting results have been reported regarding age, sex and clinical factors [6,11]. Other reasons for pre-hospital delay could be difficulties to interpret symptoms of MI because the symptoms are different from what the patient had expected [12-14] and uncertainty in how to respond to the symptoms [15].

There are also conflicting data regarding whether there are differences in pre-hospital delay in seeking care for MI between patients with and without diabetes. Some studies have found that patients with diabetes delay longer than patients without diabetes, both in STEMI (ST-elevation Myocardial Infarction) and NSTEMI (Non ST-elevation Myocardial Infarction) [7,16,17]. Others have found no differences in delay time between the groups [18,19]. Only one study investigated sex differences in pre-hospital delay among persons with diabetes, and no sex differences were found [20]. In a recent report from the Northern Sweden MONICA study, atypical symptoms were not more frequent among patients with diabetes than in patients without diabetes [21]. Analysis of patterns in pre-hospital delay in men and women with and without diabetes are important and may improve patient education aiming to reduce delay times in MI. Therefore the aim of the present study was to describe pre-hospital delay in a first MI among men and women with and without diabetes and to describe the association between pre-hospital delay time and diabetes, sex, age, symptoms and size of residential area as a proxy for distance to hospital.

## Methods

This population-based study was based on data from the Northern Sweden MONICA Myocardial Infarction Registry. The register was developed from the WHO MONICA project (MONItoring of tends and determinants in CArdiovascular disease) which started in 1985 in the northern part of Sweden. In 1995 the WHO MONICA project was ended, but in northern Sweden it continued as a local project, and it is still ongoing. In the Northern Sweden MONICA Study all incident cases of MI and stroke were registered, and repeated population surveys on cardiovascular risk factors were performed. Details of the registry have been described previously [22,23]. Briefly, the myocardial infarction registrations in the Northern Sweden MONICA registry were based on medical records, hospital discharge registers and death certificates. Strict uniform WHO MONICA criteria have been used for coding MI events [22-24]. MI diagnosis was based on symptoms and biomarkers. If only one of the two parameters were positive, then ECG analysis was included to get the final diagnosis. ECGs were examined using the Minnesota code. The biomarker, troponin, has been used since 2000 in all hospitals in Northern Sweden [22].

The diagnosis of diabetes in the Northern Sweden MONICA Registry was based on information in medical records. In this study, patients with diabetes consisted of patients with previously diagnosed diabetes. Patients who were diagnosed with diabetes during the MI event (2.2%) were included in the non-diabetes group. Pre-hospital delay was defined as the time between symptom onset and the time for “medical presence”. According to the MONICA manual, onset is the onset of the acute symptoms of MI. Medical presence is the time at which skilled health care becomes available to the patient, either in the form of medical practitioner or specially trained and equipped team of paramedics. If no medical practitioner or paramedics were present, then arrival at hospital is counted as the time of medical presence.

Variables from the MONICA registry used in this study were: “time between onset and medical presence”, diabetes, sex, age, hypertension, smoking, previous angina, size of residential area and typical-atypical symptoms (definition of typical-atypical symptoms have been described previous [21]). Age was divided into 2 groups, younger (25–64 years) and older (65–74 years). To distinguish early from late responders to MI symptoms the cut-points of < 2 h vs. ≥ 2 h were chosen. The 2-h cut-point was chosen because of the greater benefit of reperfusion therapy in STEMI patients treated within 2 h [25]. Size of residential area was divided into rural area (<1 000 inhabitants), town (1 000–15 000 inhabitants) or city (>15 000 inhabitants).

### Participants

**Figure 1:**
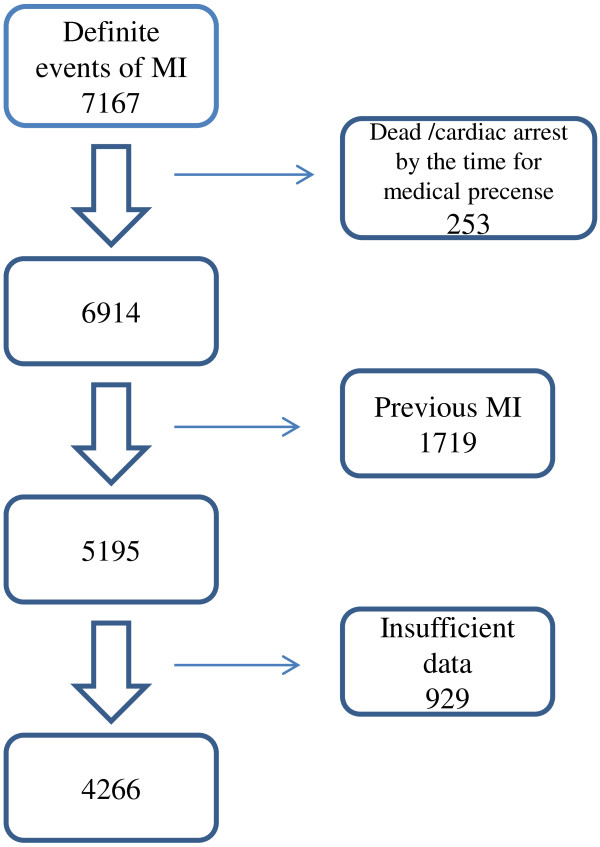
Flowchart of inclusion and exclusion of patients in the study of delay in myocardial infarction in the Northern Sweden MONICA registry between 2000 and 2008.

A total of 7167 definite events of MI were recorded in the Northern Sweden MONICA Myocardial Infarction Registry in patients 25–74 years old between 2000 and 2008 (Figure 1). Excluded were patients who were already dead or in cardiac arrest by the time they reached medical presence (*n* = 253) and patients with previous MI (*n* = 1719). Also patients coded with “insufficient data” or “not known” for time to medical presence (*n* = 929) were excluded. In the excluded observations due to “insufficient data” or “not known”, the mean age was slightly higher (63.6 year’s vs 62.8 years), the proportion of patients with diabetes was higher (19.7% vs 16.9%) and there was a higher proportion of women (32% vs 29.5%) compared with the study population. Thus, 4266 patients with a first MI were included in the analysis. 

### Statistical analysis

Continuous data were presented as means and SD, and categorical data as absolute numbers and percentages. Proportions were compared between groups using chi-square test and 95% confidence intervals for the difference. Continuous variables were compared using Student *t*-test. Multiple logistic regressions were used to estimate the association between pre-hospital delay ≥ 2 h (dependent variables) and diabetes, age, sex, size of residential area and typical/atypical symptoms (independent variables). Forced entry method was used in the multivariate logistic regression, and variables included in the model were those with significant associations (*p* < 0.05) on pre-hospital delay ≥ 2 h in the univariate analysis. Results are presented as odds ratio (OR) with 95% confidence intervals (CI). Tests of statistical significance were considered significant if *p* < 0.05*.* To test for interaction the interaction terms diabetes * sex and diabetes * age were entered in the logistic regression. The analyses in this study were performed using SPSS, version 18.0 for Windows and OpenEpi: Open Source Epidemiologic Statistics for Public Health, Version 2.3.1 [26].

### Ethics

All patients included in the MONICA myocardial infarction registry received a personal letter explaining the purpose of the registry and what to do if they did not give consent to registration. The Northern Sweden MONICA study was approved by the Research Ethics committee of Umeå University and by the National Computer Data Inspection Board.

## Results

### Clinical characteristics

The demographic and clinical characteristics for patients with and without diabetes and for the subgroups of men and women are shown in Table 1. Patients with diabetes were older (mean age 63.5 y) than patients without diabetes (mean age 61.8 y) (p<0.001). A higher proportion of patients with diabetes had hypertension and previous angina than patients without diabetes. Hypertension was more common in women with diabetes than women without diabetes. Among patients with diabetes, hypertension was more common in women than in men. There were no differences between groups regarding population size of residential area. 

**Table 1:** Clinical characteristics of patients with myocardial infarction

	**Patients with diabetes**			**Patients without diabetes**						
**Characteristics**	**Men**	**Women**	**Total**	**Men**	**Women**	**Total**				
	***n =*486**	***n =*233**	***n =*719**	***n =*2522**	***n =*1025**	***n =*3547**	***p***^***1***^	***p***^***2***^	***p***^***3***^	***p***^***4***^
**Age years, mean (SD)**	62.8 (7.9)	65.1 (7.7)	63.5 (7.9)	61.3 (8.7)	63.1 (9.0)	61.8 (8.8)	*p<*0.001	*p<*0.001	*p<*0.001	*p=*0.001
**Sex**										
Female (%)			32.4			28.9	*p=*0.060			
Male (%)			67.6			71.1				
Symptoms^*^										
-Typical (%)	92.0	91.7	91.9	93.2	90.0	92.2	*p=*0.8	*P=*0.9	*p=*0.4	*p=*0.5
Medical history/Risk factors^*^										
-Previous IHD (%)	39.2	43.9	40.8	25.7	27.4	26.2	*p<*0.001	*p=*0.2	*p<*0.001	*p<*0.001
-Hypertension (%)	59.9	70.2	63.3	34,7	48.9	38.7	*p<*0.001	*p=*0.008	*p<*0.001	*p<*0.001
-Smoking (%)	27.2	34.2	29.3	31.4	46.9	35.7	*p=*0.004	*p=*0.1	*p=*0.1	*p=*0.003
Size of residential area^**^										
-City (%)	34.4	33.9	34.2	37.2	40.8	38.3				
-Town (%)	32.7	38.2	34.5	31.9	31.3	31.7	*p=*0.1	*p=*0.3	*p=*0.5	*p=*0.081
-Rural area (%)	32.9	27.9	31.3	30.8	27.9	30.0				

*p*^1^: Comparison between patients with and without diabetes, *p*^2^: Comparison between men and women with diabetes, *p*^3^: Comparison between men with and without diabetes, *p*^4^: Comparison between women with and without diabetes.

*Missing data: Symptoms 5.2%, previous IHD 1%, hypertension 1.6%, smoking 18%.

**Rural area < 1 000 inhabitants, town 1 000–15 000 inhabitants, City > 15 000 inhabitants.

### Pre-hospital delay

More patients with diabetes had a pre-hospital delay ≥ 2 h than patients without diabetes, 64% vs. 58.% (p=0.002). The risk difference was 6.1%-points (CI 2.3-10). More women with diabetes delayed ≥ 2 h than women without diabetes (68% vs. 60%, p=0.015), risk difference 8.6%-points (CI 1.9-15.4). More men with diabetes delayed ≥ 2 h than men without diabetes (63% vs. 58%, p=0.048), risk difference 4.8%-points (CI 0.1-9.5). There was no difference in pre-hospital delay time between men and women with diabetes (Table 2). 

**Table 2:** Differences in pre-hospital delay times between men and women with and without diabetes

	**Pre-hospital delay time ≥ 2h**	***p*-value**	**Risk Difference (95% CI)**
**Patients with diabetes***n=719*	64.5%		
**Patients without diabetes***n=3547*	58.4%	*p*=0.002	6.1 (2.3-10) %-point
**Women with diabetes***n=233*	68.2%		
**Men with diabetes***n=486*	62.8%	*p=*0.150	5.5 (−1.9-12.9) %-point
**Men with diabetes***n=486*	62.8%		
**Men without diabetes***n=2522*	57.9%	*p* =0.048	4.8 (0.1-9.5) %-points
**Women with diabetes***n=233*	68.2%		
**Women without diabetes***n=1025*	59.6%	*p* =0.015	8.6 (1.9-15.3) %-point

### Factors associated with pre-hospital delay ≥ 2 h

In univariate logistic regression analysis, sex was not significantly associated with pre-hospital delay ≥ 2 (Table 3). Diabetes, older age and living in small municipalities or rural areas were all associated with longer delay. Patients presenting with atypical MI symptoms had lower risk for delay ≥ 2 h (OR 0.59, CI 0.47-0.75). In the multivariate logistic regression model diabetes, age ≥ 65, typical symptoms, living in towns or rural areas were factors associated with pre hospital delay ≥ 2 h (Table 3). The multivariate adjusted OR for atypical symptoms did not differ from the unadjusted value. There were no interactions between diabetes and sex or between diabetes and age, and they were not included in the regression model. 

**Table 3:** Univariate and multivariate logistic regression of factors associated with pre-hospital delay ≥ 2 h

	**Univariate analysis**			Multivariate analysis**		
	**OR**	**95% CI**	***p-value***	**OR**	**95% CI**	***p-value***
***Diabetes***						
No	1.0			1.0		
Yes	1.29	1.10-1.53	*p =* 0.002	1.28	1.08-1.52	*p =* 0.005
***Sex***						
Men	1.0					
Women	1.10	0.97-1.27	*p =* 0.130	*------*	*-------*	*-------*
***Age***						
25-64	1.0			1.0		
65-74	1.20	1.06-1.36	*p =* 0.003	1.22	1.07-1.38	*p =* 0.003
***Symptoms***						
Typical	1.0			1.0		
Atypical	0.59	0.47-0.75	*p <* 0.001	0.58	0.46-0.73	*p <* 0.001
Size of residential area*						
City	1.0			1.0		
Town	1.30	1.12-1.50	*p <* 0.001	1.28	1.10-1.48	*p =* 0.002
Rural area	1.55	1.34-1.81	*p <* 0.001	1.51	1.30-1.77	*p <* 0.001

^*^Rural area < 1 000 inhabitants, Town 1 000–15 000 inhabitants, City > 15 000 inhabitants.

**The multivariate analysis adjusts for all variables except for sex.

## Discussion

In our study, a higher proportion of patients with diabetes had longer pre hospital delay than patients without diabetes when suffering a first myocardial infarction. Among patients with diabetes there were no differences in pre-hospital delay time between men and women however, the largest risk difference for pre-hospital delay ≥ 2 h was between women with and without diabetes. Diabetes, older age and living in towns or rural areas were all factors associated with pre-hospital delay ≥ 2 h. Our study is unique because it includes solely patients with first myocardial infarction, and it describes pre-hospital delay among both men and women with and without diabetes.

Our results are similar to previous research, which has also found that patients with diabetes have longer pre-hospital delay than patients without diabetes [7,11,16,17]. In contrast, some studies have found no differences in pre-hospital delay between patients with and without diabetes [19,27,28]. However, those studies had fewer patients (n=140-403) [19,27], included patients with acute coronary syndrome [28], and included both first and recurrent MI [19,27,28].

In a recent report from the Northern Sweden MONICA Study, and in our present study, typical MI symptoms were common and atypical symptoms were not more frequent among patients with diabetes than in patients without diabetes [21]. Differences in typical/atypical symptoms between patients with and without diabetes could thus not be the explanation for the difference in pre-hospital delay between the groups. A possible explanation could be that patients with diabetes, like other patients with chronic illnesses, adjust to their symptoms and therefore ignore or overlook new symptoms [29,30]. In addition, the interpretation of MI symptoms as cardiac may be masked by symptoms associated with diabetes [30,31]. Vague MI symptoms such as dizziness and sweating can be interpreted as symptoms of low blood sugar level and not as symptoms of MI [30,32].

In addition to diabetes, older age and living in towns or rural areas were also factors associated with pre-hospital delay ≥ 2 h. These results are consistent with prior studies, which have also found that older age is associated with longer pre-hospital delay [7,11,16,17]. One study investigated residential area as a factor associated with pre-hospital delay. In contrast to our results, no association was found between size of residential area and pre-hospital delay > 160 min [28]. In our study, size of residential area indirectly reflects distance to hospital but probably also socio-economic differences which may partly explain differences in delay.

Presenting with atypical MI symptoms was not in our result a predictor for pre-hospital delay ≥ 2 h. This is in contrast to most previous studies, which report that typical symptoms, such as chest pain, and interpreting symptoms as cardiac in origin predict shorter pre-hospital delay [28]. Similar to our results, two studies have reported that patients with atypical symptoms had shorter pre-hospital delay [18] and no association was found between chest pain and delay [9]. Atypical symptoms such as syncope and dyspnea could be perceived with high intensity and as threatening. Fear, and experiencing symptoms as serious and/or life-threatening, have been described as factors for shorter pre-hospital delay times [33,34]. It is also possible that emotional factors such as anxiety can play an important role in the decision making process [35].

As stated earlier, shortening the time interval between symptom onset and reperfusion is crucial for reducing mortality in MI [4], and patients with diabetes have higher mortality from MI than patients without diabetes [2,3]. The longer pre-hospital delay among patients with diabetes may contribute to their higher mortality in MI compared with non-diabetics. Therefore, it is of utmost importance for health care personnel to educate patients with diabetes and to inform them about how to respond to symptoms of MI in order to shorten pre hospital delay.

The major strengths of our study are the large sample size (n=4266) and the fact that the large population-based database is controlled internally and externally for quality. Furthermore, it is a strength that all MI events are registered in the MONICA infarction registry and not only those treated in cardiology departments. To only include first MI strengthens the specificity of the findings and removes possible learning effects from a previous experience of seeking care for MI. Information about time between symptom onset and medical presence was missing in the medical records in 18% of the observations, and were thus excluded from the analysis. However; there were no major differences regarding diabetes status, gender, age and size of residential area between the excluded observations and the study population.

A limitation, however, is that information about pre-hospital delay time, in the MONICA registry was based solely on medical records. It has been discussed that other pre-hospital delay times are documented in medical records than in patient interviews [36]. Medical records are often made directly at admission to hospital, which can make it easier for the patient to remember times. Patient interviews are often made some days after arrival to hospital and therefore a risk for recall bias. It can also be difficult, both for patients and health care personnel, to delineate symptom onset of MI from prodromal symptoms, i.e. pre infarction angina before the acute MI event [37].

A further limitation is the lack of data on socio-economical status in the MONICA Study. As diabetes is more common in people with low education and both these factors are more common in rural areas, the relationships noted could be confounded. In a sensitivity analysis we studied the differences in delay time restricted to urban dwellers and found similar longer delay in diabetic subjects as for the whole group (data not shown).

## Conclusions

In summary, we found that a higher proportion of patients with diabetes have longer pre-hospital delay in myocardial infarction than patients without diabetes. There are no differences in pre-hospital delay between men and women with diabetes, the largest risk difference for pre-hospital delay ≥ 2 h is between women with and without diabetes. Diabetes, older age and living in municipal or rural areas are predictors for pre-hospital delay ≥ 2 h. Despite no differences in reported MI symptoms, patients with diabetes have longer pre-hospital delay than patients without diabetes. That indicates that the process from symptom onset to the decision to seek medical care is complex. It raises the questions of what other factors determine when to seek medical care for MI symptoms and if the decision making process to seek care differs for patients with diabetes.

## Competing interests

The authors have no competing interests to declare.

## Authors’ contributions

All authors fulfill the criteria for authorship. KHÄ, CB, UN, ME participated in the design of the study. KHÄ, CB, ME performed the statistical analysis. KHÄ drafted the manuscript, CB, UN, ME contributed in drafting and revising the manuscript. All authors read and approved the final manuscript.

## Pre-publication history

The pre-publication history for this paper can be accessed here:

http://www.biomedcentral.com/1471-2261/13/6/prepub
